# Leveraging Walking Performance to Understand Work Fatigue Among Young Adults: Mixed-Methods Study

**DOI:** 10.2196/16376

**Published:** 2020-11-13

**Authors:** Xinghui Yan, Pei-Luen Patrick Rau, Runting Zhong

**Affiliations:** 1 Department of Industrial Engineering Tsinghua University Beijing China; 2 School of Information University of Michigan Ann Arbor, MI United States; 3 School of Business Jiangnan University Wuxi China

**Keywords:** work fatigue, fatigability, walking performance, 6MWT, mobile health

## Abstract

**Background:**

Work fatigue negatively impacts personal health in the long term. Prior research has indicated the possibility of leveraging both walking parameters and perceptual measures to assess a person’s fatigue status. However, an effective and ubiquitous approach to assessing work fatigue in young adults remains unexplored.

**Objective:**

The goals of this paper were to (1) explore how walking rhythms and multiple streams of data, including reaction time, self-reports, and an activity diary, reflect work-induced fatigue in the lab setting; (2) identify the relationship between objective performance and subjective perception in indicating fatigue status and fatigability; and (3) propose a mobile-based assessment for work-induced fatigue that uses multiple measurements.

**Methods:**

We conducted a 2-day in-lab study to measure participants’ fatigue status using multiple measurements, including the stair climb test (SCT), the 6-minute walk test (6MWT), and the reaction time test. Both the SCT and the 6MWT were conducted at different points in time and under 2 conditions (measurement time, including prior to and after work, and pace, including normal and fast). Participants reported their fatigue perception through questionnaires completed before conducting walking tests and in an activity diary recorded over a week. Walking performance data were collected by a smartphone with a built-in 3-axis accelerometer. To examine the effect of fatigability on walking performance, we first clustered participants into 2 groups based on their reported mental fatigue level in the entry surveys and then compared their walking performance using a generalized linear model (GLM). The reaction time was examined using a 2-way repeated-measures GLM. We conducted semistructured interviews to understand participants’ fatigue perception after each day’s walking tests.

**Results:**

All participants (N=26; mean age 24.68 years) were divided into 2 groups—the fatigue-sensitive group (11/26, 42%) and the fatigue-nonsensitive group (15/26, 58%)—based on their mental subscores from 3 entry surveys: Fatigue Scale-14, Three-Dimensional Work Fatigue Inventory, and Fatigue Self-Assessment Scale (FSAS). The fatigue-sensitive group reported a significantly higher FSAS score in the before-work setting (t_50_=–3.361; *P*=.001). The fatigue-sensitive group covered fewer steps than the fatigue-nonsensitive group (β_1_=–0.099; SE 0.019; *t*_1_=–5.323; *P*<.001) and had a higher step-to-step time variability in the 6MWT (β_1_=9.61 × 10^–4^; *t*_1_=2.329; *P*=.02). No strong correlation between subjective and objective measurements was observed in the study.

**Conclusions:**

Walking parameters, including step counts and step-to-step time variability, and some selected scales (eg, FSAS) were found to reflect participants’ work-induced fatigue. Overall, our work suggests the opportunity of employing mobile-based walking measurements to indicate work fatigue among young adults.

## Introduction

Cognitive fatigue induced by intense or prolonged work has become a severe health issue among young adults and may result in depression and other mental conditions if not relieved in time [1]. In a study conducted by Johnston et al [2], researchers found that it was the cognitive demand rather than the physical work that led to persistent fatigue perception among nurses. The sustained mental work compressed in a short amount of time may have a severe negative impact on an individual’s well-being [2]. Fatigue is a complex reported syndrome that is associated with one’s physical and mental functionalities [3,4]. According to Enoka and Duchateau [3], fatigue has two interdependent attributes: perceived fatigability and performance fatigability. Both attributes are used to characterize the trait and state properties of fatigue [3]. The trait level of fatigue describes a person’s fatigue experienced in the preceding several days, whereas the state level of fatigue represents the changes of one’s fatigue status in response to a fatiguing task [3]. Drawing on the taxonomy of fatigability in the literature [3,4], we categorize work-induced fatigue among young adults in the working environment as a state level of fatigue, which could be measured from both perceived (subjective) and performance (objective) aspects.

To investigate both perceived and performance fatigability in current practice, researchers leverage subjective and objective measurements [3-5]. We categorize the subjective measurements of fatigue, which are usually questionnaires, into 3 genres: (1) general fatigue scales [6], (2) specific fatigue indexes (physical, mental, work, or emotional) [7-9], and (3) auxiliary diagnoses to evaluate health status, such as sleep quality and diet. In particular, the Fatigue Scale-14 (FS-14) [6], the Three-Dimensional Work Fatigue Inventory (3D-WFI) [7], and the Fatigue Self-Assessment Scale (FSAS) [8,9] have subscales evaluating physical and mental aspects. They are all valid and applicable to healthy and subhealthy populations of 18 years and older. The FSAS is specifically designed in accordance with the cultural characteristics and language habits of Chinese populations [8,9]. It has been clinically evaluated and has adequate internal consistency, with an overall Cronbach α of .953 [8,9]. The 18-item 3D-WFI identifies work exhaustion from physical, mental, and emotional dimensions [7]. Notwithstanding the effectiveness of using scales to understand perceived fatigue, such data collection methods require users to manually record experience data. Especially in field studies, recording fatigue perception upon system prompts in different situations would put a high demand on participants and cause interruptions to their ongoing work [10]. The demand placed on users points to a need to consider measuring people’s fatigue status through nonintrusive methods, such as passive sensing.

Prior research has demonstrated that physical outcome variables, for example, heart rate variability [11], reaction time (or flicker perception time) [12], and walking performance, can reflect the level of cognitive fatigue. These measurements are thought to be more reliable (less biased due to their objective nature) and less obtrusive to participants’ everyday life as opposed to self-reported data. Specifically, some variables, such as reaction time and walking performance, could be easily captured by daily mobile and wearable devices like smartphones and smartwatches. For example, Iwaki and Harada [12] designed a mobile app to measure reaction time and exploited it to infer cognitive fatigue. In addition, walking performance measurements like the 6-minute walk test (6MWT) have been widely used in prior work to indicate individuals’ physical and mental health status [13-16]. Researchers have extracted multiple walking parameters (eg, step count, speed, covered distance in the given time) to reflect one’s cognitive fatigue. For example, in a single- and dual-task 6-minute walking study with 16 young adults and 16 older participants, researchers found that only older adults’ walking performance was susceptible to mental fatigue, manifesting as an increase in their gait variability in the dual-task condition (ie, walking speed, stride length, stance time, double support time, and swing time) [17].

With the development of mobile and wearable computing technologies, the investigation of fatigue can be expanded into everyday contexts [18-23]. Prior work on fatigue measurements mainly tested these variables in the lab setting, which was limited in ecological validity. In recent years, researchers have employed walking performance measurements like the 6MWT in field studies to measure fatigability [24,25] and physical capability [15,26]. However, little research has been done to investigate how physical performance, as well as the subjective perception of fatigue, can reflect users’ state fatigue triggered by cognitive work in a natural setting. The association between individuals’ physical performance, subjective perception of fatigue, and real-life work status remains unstudied yet highly valuable. By identifying the impact of intense or prolonged work on people’s performance and their perception related to fatigability, researchers can predict people’s work performance and productivity and further make health interventions. In addition, there is also little work examining work-induced fatigue among young healthy adults, who usually do not get sufficient clinical care but face a high risk of being mentally exhausted and worn out [27]. Therefore, in order to investigate perceived and performance fatigability among young adults, we conducted a 2-day in-lab study to examine how physical performance and subjective perception could indicate young adults’ work-induced fatigue status. In particular, we aimed to answer 3 research questions: (1) How do different subjective and objective measurements indicate fatigability among young adults? (2) Is there a relationship between subjective and objective measurements of fatigability among young adults? and (3) How should we design mobile health systems that are effective and user-friendly for young adults?

To answer the 3 research questions in this paper, we designed a smartphone-based integrated measurement framework that used the 6MWT as an essential assessment to investigate Chinese college students’ and young researchers’ work fatigue. We employed reaction time and walking tests (ie, the stair climb test [SCT] and the 6MWT) as the 2 main objective measures, as well as 3 subjective scales (ie, FS-14, FSAS, and 3D-WFI). We used these measures to compare the performance and perception data between a fatigue-nonsensitive group and a fatigue-sensitive group, which were grouped by participants’ reported fatigue level preceding their participation in the study.

Overall, the contributions of this work are threefold. First, we demonstrate the feasibility of using selected walking parameters (ie, step count and step-to-step time variability) to indicate work fatigue among young healthy adults. Second, we investigate the relationship between perceived fatigue and performance measurements of fatigue and their capabilities of reflecting work-induced fatigue. Third, combining perceived and performance measurements of work-induced fatigue, we propose a mobile design framework, along with 3 design implications.

## Methods

### Overview

The goal of our study was to investigate performance fatigability and perceived fatigability among young adults who conduct intense cognitive work daily. Moreover, we aimed to understand the relationship between subjective and objective measurements surrounding the state property of fatigability. To achieve these goals, we conducted a 2-day in-lab study to examine participants’ physical performance in different conditions, varying the test’s time of occurrence and walking pace. By implementing walking tests at different times, we studied how participants’ fatigue changed as their work proceeded.

### Participant Recruitment

We randomly selected 26 participants (14 women and 12 men; mean age 24.68 years, SD 4.34) out of 49 volunteers from Tsinghua University who met the screening requirements. To meet the eligibility criteria of this experiment, participants had to (1) have no walking disabilities, (2) work at least 6 hours per day, and (3) have not been involved in workout activities during work. All participants reported high research pressure and work stress. The average work duration per day was 9.10 (SD 1.59) hours during the last 3 months, and the average self-reported work-induced fatigue score (within the last 3 months) was 7.80 (SD 1.35) out of 10. We ensured that the selected participants met our research criteria based on their activity diaries and responses to the questionnaires. In the study, participants were free to schedule their personal work and time to relax. The actual measurement time was dependent on participants’ work schedules and therefore varied from person to person.

### Selecting Fatigue Measurements

In this study, we applied both subjective and objective fatigue measurements. In terms of subjective measurements, we referred to the literature and selected the FS-14 [6], FSAS [8,9], and 3D-WFI [7]. These measurements cover the examination of general fatigue level and work-related fatigue level in the mental subscales. The selection of objective measurements was based on the criteria that (1) the investigated data could be captured in the working environment, (2) the measurements were not obtrusive to participants’ regular work, and (3) the measurements could reflect a person’s real-time or nearly real-time fatigue state. Based on the criteria, we selected walking performance and reaction time for investigation. First, walking performance has been demonstrated to be a valid physical measurement that indicates older adults’ cognitive fatigue [17], but it has not been validated in young adults. Moreover, the measurement of walking performance could be conducted by smartphones without additional sensors. Among various walking performance tests, we selected the 6MWT because the short duration was thought to be less intrusive on a person’s daily work and more acceptable. Second, reaction time has been widely used in prior work to measure cognitive fatigue in situ [18]. It can be executed within a minute and implemented on personal devices such as laptops and smartphones [18]. In our study, we were interested in examining how these variables reflect fatigability and how they correlate with one another.

### Experiment Design and Procedure

The goal of our study was to explore the effect of work-induced fatigue on multiple variables, including walking performance, reaction time, and subjective perception. We first grouped our participants into 2 groups (fatigue-nonsensitive group and fatigue-sensitive group) based on their perceived work fatigue level in the preceding 6 months. We then conducted a 2-day in-lab walking test to measure participants’ walking performance before and after their work time. The walking tests were conducted under 2 settings (stair climbing and flat-ground walking) and at 2 different paces (normal and fast pace), resulting in 4 walking conditions. Each participant was required to perform the walking tests under each condition before and after work. Thus, the number of computing instances for a single participant was 16 (2 days × 2 times × 2 paces × 2 settings). In addition to the walking test, we applied a reaction time test (RTT) to assess participants’ fatigue performance. For perceptual measures, participants were required to fill out a set of scales at the beginning of each day’s tests, including FS-14, FSAS, and 3D-WFI (only applied to the after-work test). The questionnaires were used to study participants’ perceived work fatigue and its change throughout the day. In addition, we invited participants to record their daily activities during the week of the study. This was to help us investigate participants’ work-related schedules, which might affect their fatigue perception and performance.

**Figure 1:**
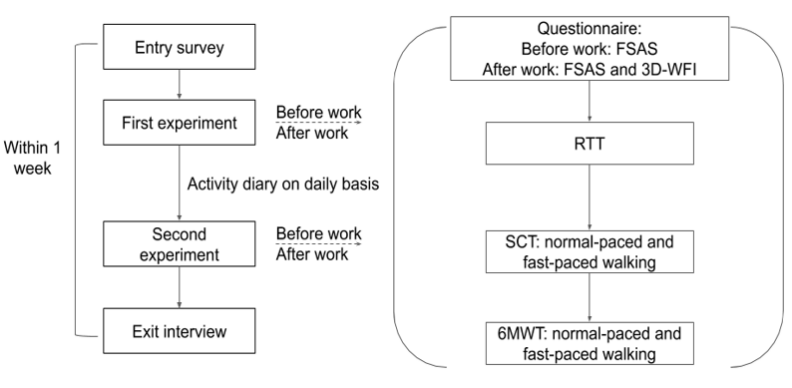
Experiment procedures. 3D-WFI: Three-Dimensional Work Fatigue Inventory; 6MWT: 6-minute walk test; FSAS: Fatigue Self-Assessment Scale; RTT: reaction time test; SCT: stair climb test.

The experiment procedure is shown in Figure 1, with specific items noted. On each visit, upon participants’ arrivals, they were asked to first fill out a questionnaire and then conduct the RTT. Next, we invited participants to conduct the SCT and 6MWT at normal and fast paces. Participants were not allowed to pause between the SCT and 6MWT. There were 16 steps (15 cm in height for each stair), and the participant was required to walk up and down 2 levels of the building, resulting in 64 steps in total. The flat-ground walking test had no constraints and participants walked to the end and back of a 75-m corridor. The start point and end point were clearly marked on the ground. After the walking tests, we held a brief semistructured interview with our participants, asking questions such as “How are you feeling right now after taking the walk?” and “Did you have any difficulties in the fast-paced walking?”

### Device

**Figure 2:**
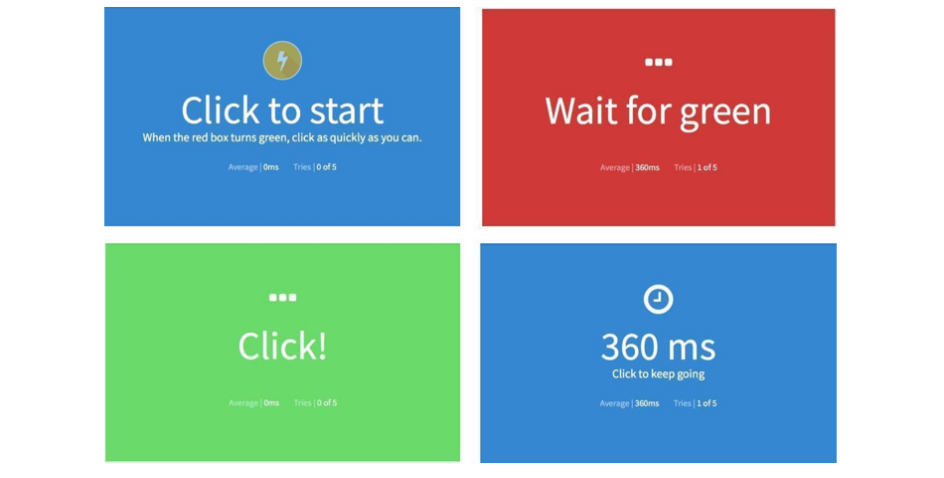
Reaction time test user interface [28]: (1) preparation of the test and instructions, (2) “wait for green” text alerting users that test has begun, (3) appearance of green and the text hint “Click!” and (4) result shown to users, with average result attached.

**Figure 3:**
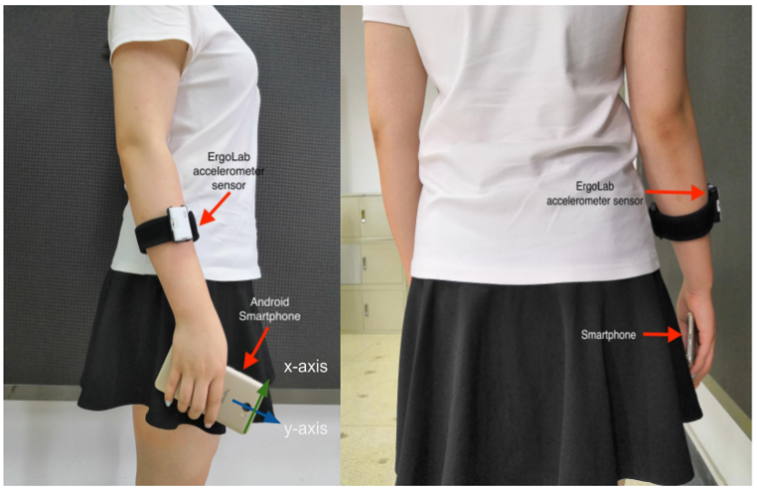
Participants wore the ErgoLab sensor and carried the Huawei smartphone during the walking tests.

A set of digital questionnaires were offered to participants, and they were required to take an online computer-based RTT prior to each walking test. The RTT that we used in the study was developed by Human Benchmark (see Figure 2) [28], and we adapted the colored block into a full screen to avoid other website components disrupting participants. Participants were asked to click as fast as possible when they perceived that the red block (ready mode) turned green (react mode). To measure walking performance, we used a benchmark sensor system (ErgoLab; Beijing King Far Corp) and an Android smartphone (Huawei 5C with 3-axis accelerometer sensors built in) (see Figure 3). The ErgoLab accelerometer sensor (frequency of 64 Hz) was placed on the participants’ right wrist, the same side as the hand holding the smartphone. Data from the sensor were used as ground truth data for smartphone sensor data processing and analysis. In their right hands, participants carried an Android smartphone with a mobile app installed to collect and preprocess the walking data.

### Walking Signal Processing

**Figure 4:**
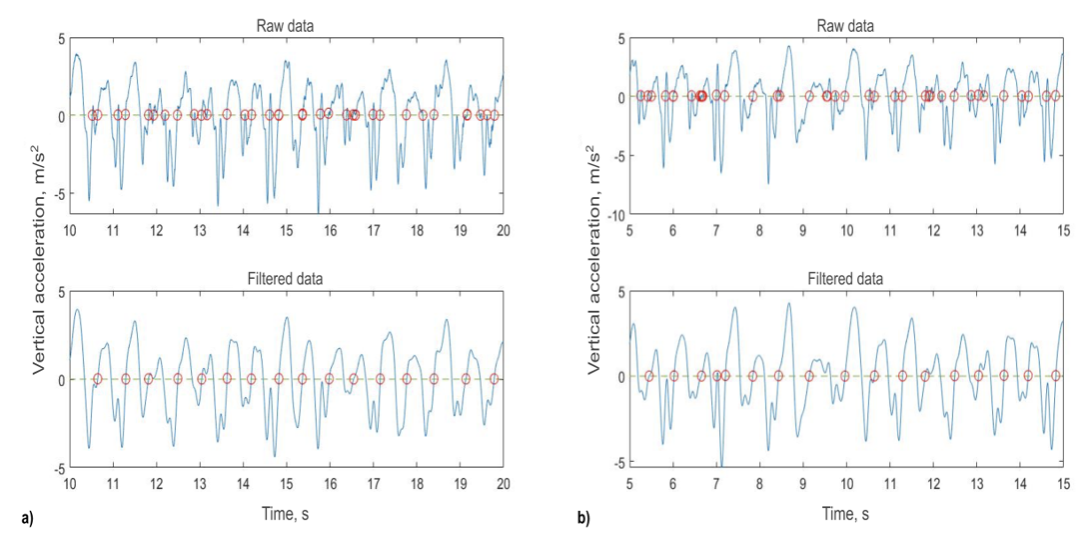
We adopted the x-axis for signal processing and presented two 5-second computing examples.

The sampling frequency of the smartphone built-in sensor was around 30 to 50 Hz*.* The unequal sampling frequency was caused by the delay of the software reading in the accelerometer data. For signal processing, we referred to the algorithm proposed by Capela et al [29], which offered a solution to analyze the calibration-free 6MWT data [29]. The signal processing was conducted through MATLAB (MathWorks Inc). We first resampled the data to 30 Hz to address the unequal sampling frequency issue*.* We then applied a fourth-order zero-lag Butterworth low-pass filter using 4 Hz as the cutoff frequency [29]. We applied a moving window of 4 seconds to analyze the vertical acceleration data and identified positive zero-crossings for step detection (see Figure 4). In Figure 4, all positive zero-crossings were labelled and used to determine the step duration. Based on the study by Capela et al [29], we set the thresholds of step duration time as between 0.4 seconds and 0.7 seconds. We also set the rule that the time change between 2 consecutive steps should not exceed 20%. Otherwise, we would drop the positive zero-crossing data and take the average of the prestep and poststep time duration as the current step time. As we instructed our participants to hold the smartphone in their hand, we processed and analyzed both the x-axis and y-axis signals (the z-axis signal was uncorrelated with the walking direction). The results from both the x-axis and y-axis signals were compared with ground truth data. We found that the results from the x-axis signal had higher accuracy in this study. Therefore, we adopted data from the x-axis for analysis.

### Statistical Analysis

The mental subscores of the 3 entry surveys were first rescaled to 0 or 1 and used to divide all the participants into 2 groups. The clustering performance was evaluated using a silhouette coefficient and a 2-tailed Welch *t* test due to unequal variance and unequal sample sizes. The silhouette score was used to measure how well data points were matched to the clustered group. We applied a repeated-measures generalized linear model to compare the step counts between the fatigue-sensitive group and fatigue-nonsensitive group. We chose quasi-Poisson regression to model the step count data, as it is generally used for modeling count data and performs better when there is overdispersion in the model [30]. It is also advantageous in comparing the performance of 2 groups with unequal sizes. We applied dummy coding for the grouping variable (fatigue-nonsensitive group: 0; fatigue-sensitive group: 1), time (before work: 0; after work: 1), and pace (normal: 0; fast: 1). We used a linear mixed-effects model to analyze step-to-step time variability. Linear mixed-effects models do not require the data to be independent (different walking trials of a person might be intercorrelated) and can account for both fixed and random effects. In the models for both step count and step-to-step time variability, we had 3 categorical variables, which were time, pace, and group membership, and participants were treated as random effects. A 2-way repeated-measures generalized linear model with group membership and time as independent variables was applied to analyze the reaction time because of the unequal sample size in the 2 clustered groups. For correlation analysis, we applied Pearson correlation analysis. It was used to investigate the relationships between walking performance and the fatigue perception information that was acquired by questionnaires. We used the correlation coefficient *r* to determine the strength of the correlation between two variables (strong correlation was >0.8). Statistical significance was defined as *P*<.05 for all tests. In addition to the quantitative results, we also present activity diary data and key findings from the interviews.

## Results

### Generating Group Memberships and Analyzing Subjective Scales

In Table 1, we present the subjective scale data collected during the entry survey and the 2-day in-lab sessions. The FS-14 is a yes-or-no questionnaire, so in our analysis, it was first adapted into the 1 or –1 rating form. We grouped all participants into 2 groups (group 1: n=11; group 2: n=15; silhouette coefficient=0.429). Results showed that participants in group 1 reported significantly higher fatigue related to cognitive work on all 3 scales (see Table 2). We classified group 1 as the fatigue-sensitive group, that is, participants who were more likely to perceive exhaustion and tiredness due to cognitive work. In contrast, participants in the fatigue-nonsensitive group were relatively less likely to perceive fatigue under similar workloads.

**Table 1:** Subjective scale performance of all participants.

Scales		FS-14^a^	3D-WFI^b^ (total)	3D-WFI (mental)	FSAS^c^ (total)	FSAS (mental)	
Score, range		–14 to 14	0 to 72	0 to 24	0 to 56	0 to 32	
Type		Mental	Total score	Mental	Total score	Mental	
Overall, mean (SD)^d^		3.00 (1.06)	49.42 (11.88)	18.15 (4.16)	36.46 (12.26)	9.77 (3.49)	
**Day 1**							
	Before work, mean (SD)	N/A^e^	N/A	N/A	30.95 (9.59)		21.13 (5.82)
	After work, mean (SD)	N/A	57.60 (13.57)	19.88 (4.87)	39.36 (9.21)		23.24 (5.03)
**Day 2**							
	Before work, mean (SD)	N/A	N/A	N/A	31.88 (8.82)		21.04 (5.32)
	After work, mean (SD)	N/A	49.96 (11.22)	17.81 (3.94)	37.50 (9.62)		22.77 (5.82)

^a^FS-14: Fatigue Scale-14.

^b^3D-WFI: Three-Dimensional Work-Fatigue Inventory.

^c^FSAS: Fatigue Self-Assessment Scale.

^d^The overall mean was the average score of the measurements that participants took before performing all the tests.

^e^N/A: not applicable.

**Table 2:** The *t* tests are performed on mental subscores collected from the FS-14, FSAS, and 3D-WFI in entry surveys to examine the clustering performance.

Scale	FS-14^a^ (mental)	FSAS^b^ (mental)	3D-WFI^c^ (mental)
Fatigue-sensitive group, mean (SD)	–5.40 (0.47)	7.47 (0.91)	21.27 (0.47)
Fatigue-nonsensitive group, mean (SD)	–1.00 (0.62)	–3.55 (0.77)	15.87 (0.49)
*P* value	<.001	<.001	<.001
*t* test (*df*)	–5.18 (22.27)	–6.01 (18.80)	–4.22 (21.40)

^a^FS-14: Fatigue Scale-14.

^b^FSAS: Fatigue Self-Assessment Scale.

^c^3D-WFI: Three-Dimensional Work-Fatigue Inventory.

**Figure 5:**
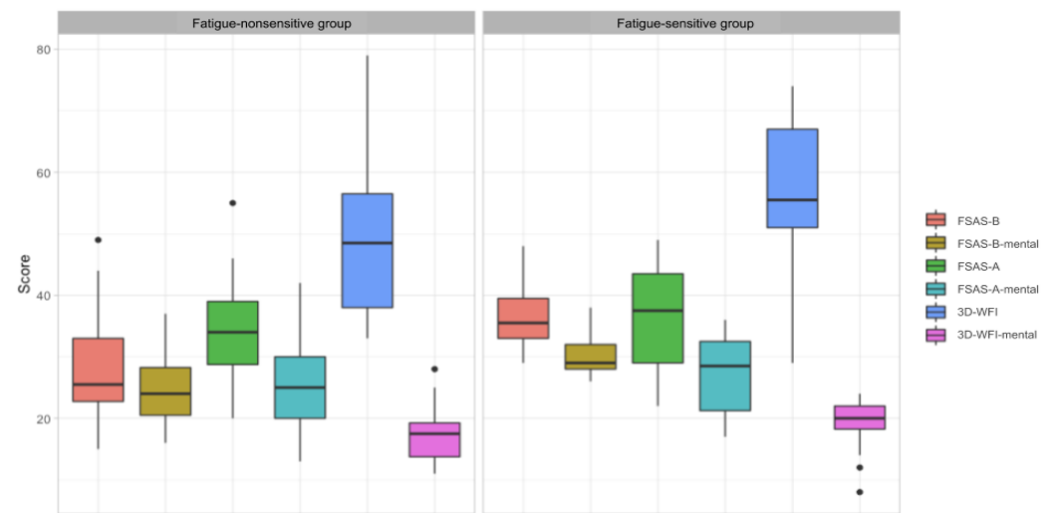
Participants reported their fatigue perception using the 3D-WFI and FSAS in the 2-day in-lab study. A: after work; B: before work; FSAS: Fatigue Self-Assessment Scale; 3D-WFI: Three-Dimensional Work Fatigue Inventory.

The perception data in Figure 5 presents participants’ responses to the 3D-WFI and the FSAS (before and after work) in the 2-day lab study. The *t* tests performed on each scale showed that the fatigue-sensitive group reported significantly higher scores on both the before-work FSAS (*t*_50_=–3.361; *P*=.001; 95% CI –14.27 to –3.50) and the before-work FSAS mental (*t*_50_=–3.30; *P*=.002; 95% CI –8.91 to –2.11) compared with the fatigue-nonsensitive group. No significant difference was found in terms of the change in responses to the FSAS.

### Reaction Time

Participants conducted 5 trials for each RTT, resulting in 520 trials in total (mean 313.47, SD 72.17 milliseconds). We did not exclude the maximum or minimum data unless participants claimed that they had difficulty using the system. There was no significant difference between the average reaction times (in milliseconds) in the fatigue-sensitive group (before work: mean 295.63, SD 37.99; after work: mean 310.83, SD 29.87) and the fatigue-nonsensitive group (before work: mean 306.33, SD 47.78; after work: mean 308.39, SD 53.33). However, from before work to after work, the average reaction time variance of all participants was significantly increased by 9.15 milliseconds (*t*_25_=–2.31; *P*=.03).

### Walking Performance

**Figure 6:**
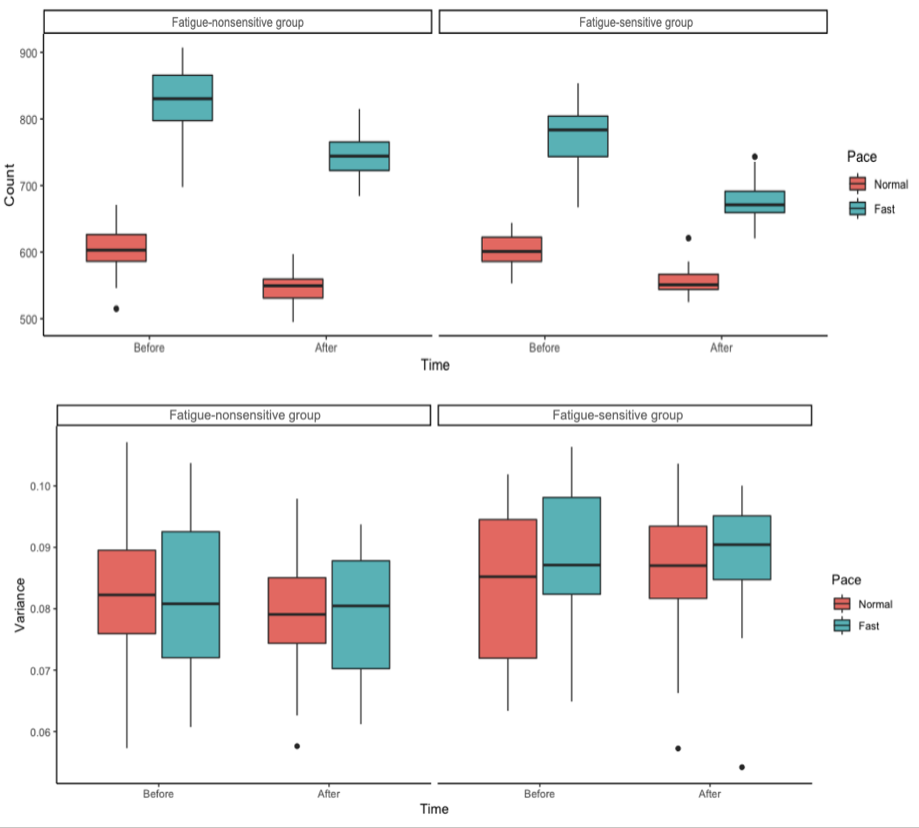
Above: Participants’ step counts in the 6-minute walk test under different conditions. Below: Participants’ step-to-step time variability in the 6-minute walk test under different conditions.

In Figure 6, we present the step count data and step-to-step time variability of the 6MWT for the 2 groups.

Results from the 2 models showed that group membership (β_1_=–0.099; SE 0.019; *t*_1_=–5.323; *P*<.001), time (β_2_=–0.102, SE=0.016; *t*_1_=–6.370; *P*<.001), and pace (β_3_=0.309, SE=0.018; *t*_1_=17.289; *P*<.001) all had a significant effect on step counts in the 6MWT. There was also an interaction effect between group membership and pace (β_5_=0.119, SE=0.028; *t*_1_=4.240; *P*<.001) on step counts in the 6MWT. This means that the change in step counts from normal to fast pace was significantly higher in the fatigue-nonsensitive group compared with the fatigue-sensitive group regardless of the measurement time. In terms of the step-to-step time variability, only group membership had a significant effect on step-to-step time variability (β_1_=9.61 × 10^–4^; *t*_1_=2.329; *P*=.02). This means that, overall, compared with the fatigue-sensitive group, the fatigue-nonsensitive group could maintain more stable step rhythms within the 6 minutes. No significant difference was observed in the SCT under different conditions.

### Relationship Between Subjective and Objective Measurements

Overall, there was not a strong correlation between subjective and objective measurement variables. There was a moderate correlation between average cadence (natural and fast-paced walk after work) and the 3D-WFI mental subscore (*r*=0.46; *P*<.001). However, step-to-step time variability during after-work walk tests did not show a significant correlation with the 3D-WFI mental subscore. The change in FSAS scores during the day had a moderate correlation with step-to-step time variability in the after-work trials (*r*=0.40; *P*=.004).

### User Interview

We did a brief semistructured interview after each walking test with participants. Under most conditions, most participants (20/26) reported that they felt refreshed after taking a walk, especially after the fast-paced walk in the before-work experiment, yet they had not expected such a positive outcome. On the contrary, nearly all participants (22/26) reported that they felt exhausted during the after-work trials. Particularly, they perceived more difficulties in keeping the initial pace during the after-work fast-paced trials compared with the before-work trials. Interestingly, participants seemed to have anticipated their walking performance before taking the walking tests. For example, nearly half of the participants (12/26) told us that they had anticipated their unsatisfactory walking performance in the after-work fast-paced trials.

In addition, we found that during the walking experiment, over half of the participants (15/26) expressed their fatigue perception, starting from around the fourth minute. For example, participant 5 told us, “I feel that I cannot walk faster now.” Moreover, when participants associated their fatigue perception with daily activities, they could tell the reasons they were tired or exhausted. For instance, participant 7 reported, “I am feeling very exhausted right now, probably because I did not take a nap at noon.” Similarly, 3 participants reported that they had done much repetitive work during the afternoon, resulting in their perceived mental fatigue.

### Activity Diary

**Figure 7:**
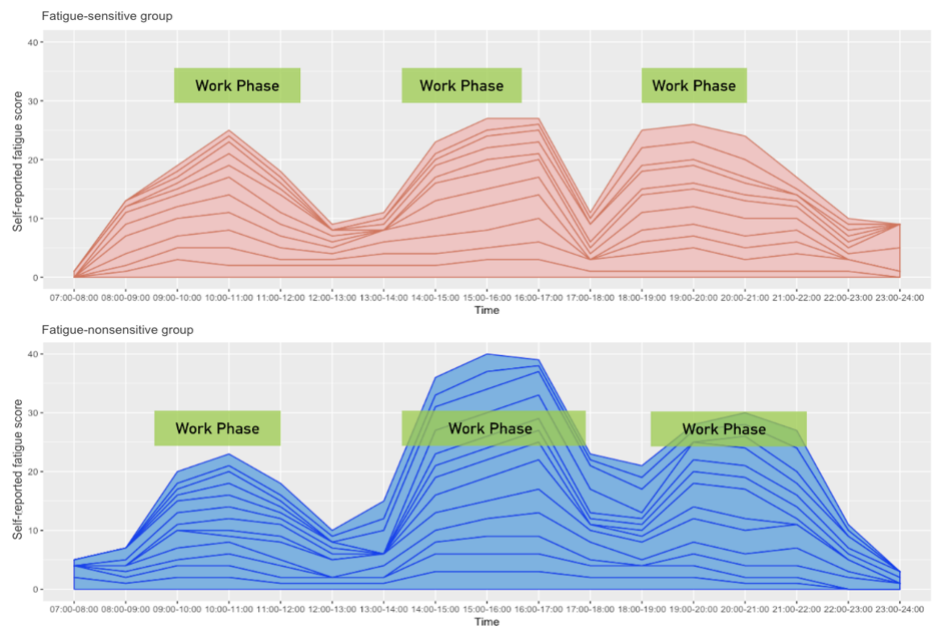
We visualized the self-rated perceived fatigue collected from the activity diary and the average work time duration for the 2 groups.

Participants were assigned an Excel (Microsoft Corp) template and asked to record their ongoing activities, rate in-the-moment fatigue perception, and annotate the activity if necessary. This task was performed to examine participants' self-reported work fatigue status and its association with daily activities. We used a 6-point rating scale for participants to rate their work fatigue perception (none, little, some, medium, severe, or very exhausted). In Figure 7, we visualized the 2 groups’ average work fatigue scores over the week. We used green boxes to label the overlapped work phases for all participants.

Overall, the trends of the 2 groups were similar, with 3 notable peaks of work fatigue at around 10:00 AM to 11:00 AM, 3:00 PM to 4:00 PM, and 8:00 PM to 9:00 PM. Interestingly, we found that the fatigue-nonsensitive group reported higher self-rated fatigue perception in the afternoon and evening, but were able to keep working 1.5 times longer on average compared with the fatigue-sensitive group. This might be because the fatigue-sensitive group tended to perceive work fatigue easier and thus might have reduced their work time due to the fatigue perception. In this regard, the fatigue-sensitive group reported lower fatigue perception compared with the fatigue-nonsensitive group.

### System Improvement

**Figure 8:**
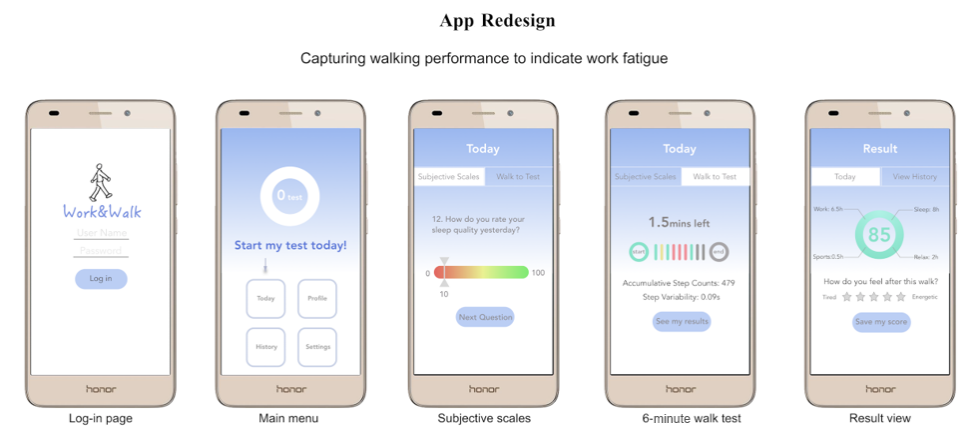
We redesigned and proposed a mobile app framework based on the results from our study.

In light of our study results, we determined the measurements that were indicative of users’ fatigue perception. In our proposed system, there are three main features: walking tests (not limited to the 6MWT), subjective questionnaires (eg, the FSAS), and activity diary logging, where participants are expected to record daily activities and their fatigue level. To reduce the length of the questionnaires, researchers could only include the mental subquestionnaires in the system. We redesigned and sketched the mobile app (see Figure 8), which contains the added selective questionnaires and activity diary not included in its original version.

## Discussion

### Principal Findings

In this paper, we reported findings from an in-lab study with young adults that explored effective measurement methods for revealing work-induced fatigue perception and performance. We leveraged multiple objective and subjective measurements, with a focus on walking performance, to investigate the best method with which to indicate young adults’ fatigue status. Moreover, we preliminarily studied how performance fatigability and perceived fatigability correlated with each other by comparing subjective scales and walking performance.

Findings from this study showed that in general, participants took significantly fewer step counts after work compared with before work. Overall, the fatigue-nonsensitive group took significantly more steps compared with the fatigue-sensitive group. Interestingly, regardless of the measurement time, we found that the change in step counts from normal pace to fast pace was significantly higher in the fatigue-nonsensitive group compared with the fatigue-sensitive group. This suggested that the fatigue-nonsensitive group showed better physical capabilities when asked to modify their walking pace. In terms of the step-to-step time variability, our findings showed that the fatigue-nonsensitive group could better maintain their walking rhythms compared with the fatigue-sensitive group. In our study, the step time variability reflected how much a participant’s step frequency was impacted by the energy exertion during the 6MWT. We think that this was pertinent to a person’s fatigability, which means that under the same physical activity exposure, participants who were more sensitive to fatigue were more likely to decrease their walking frequency in response to the increased fatigue perception. This contrasts with a previous study measuring young participants’ and older participants’ walking performance under single-task and dual-task conditions [17], as our study shows that young participants increased their gait variability over a day of work. One possible explanation is that in our study, all participants perceived cognitive workloads in a relatively naturalistic setting, and after a day of work, their fatigue level was significantly increased, especially for participants in the fatigue-sensitive group.

In reaction time, we only observed a significant change caused by the time of measurement, which means that both the fatigue-sensitive group and the fatigue-nonsensitive group had an increase in cognitive fatigue after work but did not significantly differ from each other. This might be caused by the experiment design, in which all of our participants might have become more alert when researchers asked them to conduct the RTT. Hence, we could not conclude that RTT was not an effective measurement method for identifying young adults’ fatigability. In general, correlation analysis between subjective and objective measurements did not yield strong correlations. However, we observed a moderate correlation relationship between the step counts and the 3D-WFI mental subscore (*r*=0.46; *P*<.001). The activity diary and user interview data after each walking test contributed to our understanding of work-induced perceived fatigue in young populations. Drawing on these findings, we sketched a mobile framework to study work-induced fatigue perception daily. In the “Design Implications” section, we propose 3 design implications for future work.

### Design Implications

#### Using Walking Performance Data to Identify People With a Higher Work-Induced Fatigue Level

The relationship between performance fatigability and perceived fatigability has gained growing research attention [3,4,31,32]. Among various physical outcome variables (eg, heart rate data, electroencephalography), walking is a ubiquitous human activity in everyday life and can be captured through smartphone or smartwatch built-in sensors. Prior work has demonstrated the feasibility of measuring walking performance to indicate fatigability in an older population [31]. For example, researchers found that covered distance was indicative of perceived fatigability [31]. In another study, the progression of fatigability had an effect on walking performance (eg, covered distance) during the 6MWT [32]. Building on prior work, we investigated walking performance and assessed its relationship with perceived fatigability in a different context—through work-induced fatigue among young adults. We believe that studying this population is highly valuable because the younger population undergoes high pressure in the working environment but has received less attention regarding their work-induced fatigue. In this regard, our study contributes to the investigation of fatigue among young adults. First, on the group level (fatigue-nonsensitive group and fatigue-sensitive group), we found that compared with the fatigue-nonsensitive group, the fatigue-sensitive group covered fewer steps in all the testing conditions and performed more poorly in maintaining their walking rhythms, which manifested as having larger step-to-step time variability. Our findings suggest that step count and step-to-step time variability could be used to indicate young adults’ fatigue status. This further implies the opportunity of leveraging walking measurements to signal perceived fatigability for this research population.

#### Leveraging Prompted Assessments to Track Work-Induced Fatigue Daily

In our study, we designed 2-paced 6MWTs to be required at 2 time points related to a person’s work schedule. The 6MWT is a physical performance measurement that has been extensively used in previous research quantifying individuals’ physical and cognitive exertion [31-33]. It is generally believed to be safer, easier to administer, and more reflective of the activities than other walk tests [33]. In the literature, there has been a growing trend in applying mobile technologies (eg, smartphones) to implement the 6MWT in natural settings. For instance, Brooks et al [33] developed a self-administered 6MWT mobile app and tested its usability among patients with congestive heart failure about three times a week over 2 weeks. Building on prior work, we asked participants to do a 6MWT in different conditions to capture performance changes that were subject to the measurement conditions. Findings from our study show that participants’ fatigue status had an effect on their walking performance. This points to an opportunity to introduce brief in-lab assessments (eg, the 6MWT) conducted at different conditions into participants’ daily lives. Researchers could use mobile apps to implement a research protocol similar to an in-lab study, for example, by measuring cognitive fatigue before and after being exposed to a period of cognitive work. However, in contrast to the lab setting, where researchers usually adopt a uniform cognitive task for all participants, the real-world setting has more complexity, as working status varies from person to person. In this regard, our work suggests that researchers could engage participants in recording their daily activities and rating their perceived fatigue level. In addition to this, researchers could also consider leveraging context and context awareness to identify when the user is about to work or has completed a day of work. In detecting a key event related to work, the mobile system could prompt the user to conduct a brief walking test, such as the 2MWT [32] or 6MWT, depending on the user’s availability and ongoing activities. By prompting a user to do brief walking tests at different time points in a day, researchers would be able to analyze the user’s walking performance and its association with their behavioral context, which helps better identify triggers for the user’s fatigue status. Furthermore, similar to our experiment design, in future work, researchers may instruct users to conduct walking tests at different paces. Findings from this study show that the difference in step counts between fast-paced and normal-paced walking tests could be a variable that signals fatigue status. We think that designing such multicondition prompted walking assessments would help capture the nuanced change in one’s fatigue status.

#### Integrating Multiple Measurements to Gain a Holistic View of Work-Induced Fatigue

According to prior work, fatigability is a phenotype characterized by the relationship between an individual's perceived fatigue and the activity level with which the fatigue is associated [4,34]. Fatigability is largely unexplored among younger populations, which means that there are no clinically validated metrics to derive the fatigability score for young adults using mobile sensing methods. This work acts as an initial step to explore the parameters that may have the potential to reveal young adults’ fatigability. Findings from this study show that measurements, including the 3D-WFI, FSAS mental score (its change during the day), and walking performance, can help differentiate the fatigue-sensitive group from the fatigue-nonsensitive group. Interestingly, in our study, there were also seemingly contradictory results. For example, the fatigue-nonsensitive group reported a higher perceived fatigue score in the activity diary compared with the fatigue-sensitive group. We think that this finding enriches the notion of fatigability among young adults. Although the fatigue-nonsensitive group reported higher fatigue perception compared with the fatigue-sensitive group, they could work longer and seemed to be more capable of bearing fatigue perception. The activity diary also enabled us to understand daily activities that might trigger or have an impact on one’s fatigue status. Taken together, our work points to the need for understanding fatigue status in a natural setting from multiple perspectives, such as objective performance measurements (eg, 6MWT and RTT) and self-reports, as well as activity data in context. Combining multiple sources of data available from smartphones, researchers may build a holistic view of work-induced fatigue and fatigability. In this respect, our study has contributed to several variables (eg, step count and step-to-step time variability) that are valuable for future investigation.

### Limitations

Our work has several limitations. First, due to the in-lab experiment setting, our study could not fully represent ecological validity. However, to probe one’s fatigue level, we adjusted each participant’s testing time according to their work schedule. Second, the enrolled participants were all college students or research workers, which might not be able to represent the general young adult population. Third, we only used the 3-axis accelerometer signals for analysis due to device capability, and we instructed participants to hold the phone in their hand while walking. A location-independent mobile system is needed in the future to enable the field investigation. Lastly, we applied a dichotomous classification of fatigue perception for all participants. For a more precise health-tracking purpose, a fine-level classification of fatigue levels for users would be highly valuable. Overall, this study acts as an initial step in investigating relationships between walking rhythms and work-related fatigue. Going forward, we plan to conduct a longitudinal field study to explore the effect of fatigability on multiple outcome variables.

### Conclusion

In this paper, we conducted an in-lab experiment to investigate how fatigue elicited by daily work could be captured through multiple measurements. Findings showed that there was a significant difference in walking performance (ie, step count and step-to-step time variability) and FSAS scores between the fatigue-sensitive group and the fatigue-nonsensitive group. The fatigue-sensitive group was more vulnerable to fatigue perception and less productive in daily work. Overall, our study paves the way for future work studying work-induced fatigue among young adults and designing mobile systems to capture nuanced changes in fatigue status.

## Abbreviations

3D-WFI: Three-Dimensional Work Fatigue Inventory
6MWT: 6-minute walk test
FS-14: Fatigue Scale-14
FSAS: Fatigue Self-Assessment Scale
RTT: reaction time test
SCT: stair climb test
